# Pitfalls and capabilities of various hydrogen donors in evaluation of peroxidase-like activity of gold nanoparticles

**DOI:** 10.1007/s00216-016-9976-z

**Published:** 2016-10-08

**Authors:** Marcin Drozd, Mariusz Pietrzak, Paweł G. Parzuchowski, Elżbieta Malinowska

**Affiliations:** 1 Department of Microbioanalytics, Institute of Biotechnology, Faculty of Chemistry, Warsaw University of Technology, Noakowskiego 3, 00-664 Warsaw, Poland; 2 Faculty of Chemistry, Warsaw University of Technology, Noakowskiego 3, 00-664 Warsaw, Poland

**Keywords:** Catalytic activity, Gold nanoparticles, Peroxidase mimetics, Nanozyme

## Abstract

Catalytic nanomaterials, widely used as substitutes of peroxidase, exhibit unique properties, which are unattainable for native enzymes. However, their activity is usually examined by means of substrates developed and methods standardized for horseradish peroxidase (HRP). The aim of the presented work was to determine the scope of usefulness of chromogenic substrates for gold nanoparticle (AuNP) activity studies under conditions which significantly extend beyond the activity range of a native HRP. The applicability of chromogens such as 3,3′5,5′-tetramethylbenzidine (TMB), *o*-phenylenediamine (OPD), 2,2′-azino-bis(3-ethylbenzothiazoline-6-sulfonic acid) (ABTS) beyond the typical range of pH, and for the samples of high concentration of hydrogen peroxide was examined. The conducted research confirmed the usefulness of ABTS and TMB in acidic media (pH 2.5–3.5). At the same time, potential interferences from chloride anion, unobservable for HRP-based assays, were indicated. Moreover, a number of potentially useful hints concerning relations of concentration of substrates and catalyst for aromatic amine oxidation (TMB and OPD) were proposed. By increasing the concentration of chromogens and thanks to assuring the relatively low conversion of the reaction, the stability of TMB and OPD oxidation product was improved even in acidic media. The comparative studies of H_2_O_2_ affinity to the surface of AuNPs in the presence of various hydrogen donors underlined the superiority of phenolic compounds over aromatic amines and ABTS in the case of the samples of relatively low H_2_O_2_ concentration. This work highlights some improvements in the methods of HRP-like activity characterization of NPs. It provides a critical analysis of the major challenges, which may emerge in a case of bioanalytical assays employing the catalytic nanoparticles as labels.

## Introduction

An optical detection of products generated by means of a catalytic oxidation in the presence of hydrogen peroxide has been widely utilized in bioanalytical systems. Such an indirect approach of catalytically amplified signal generation has gained popularity in the case of immunoassays and biosensors [1–5]. Numerous analytical methods based on the detection of H_2_O_2_ as the intermediate product of the catalytic oxidation of clinically relevant analytes such as glucose or ascorbic acid have been also developed [6–10]. For these purposes, the native enzymes from the class of oxidoreductases, e.g., horseradish peroxidase (HRP), were most commonly applied. The advantages of HRP, besides its high activity, include a low substrate specificity and facility of conjugation to bioreceptors [11].

Among the chromogenic substrates used as hydrogen donors in combination with H_2_O_2_ and HRP, 2,2′-azino-bis(3-ethylbenzothiazoline-6-sulfonic acid) (ABTS), phenolic compounds coupled with 4-aminoantypirine (4-AAP), catechol derivatives (e.g., dopamine, pyrogallol, bromopyrogallol red (BPR)), azo-dyes (eriochrome black T), and aniline derivatives (3,3′,5,5′-tetramethylbenzidine (TMB), *o*-phenylenediamine (OPD), *o*-dianisidine) can be distinguished [6, 7, 12–16]. It should be pointed out that they differ in solubility and susceptibility towards oxidation, and the efficiency of their oxidation is highly dependent on pH. Moreover, one should remember that the products of their oxidation are characterized with different optical properties.

The use of enzymes as catalytic labels of biomolecules imposes restrictions on the composition of the medium and conditions of the catalytic reaction. The systems based on native HRP, due to its protein origin, are sensitive to pH lower than 3.5 and higher than 9.0 or/and high concentration of hydrogen peroxide. Typically, for all enzymes, they are also susceptible to the inhibition caused by an increased temperature or presence of heavy metal ions and other substances such as thiols and azides [12, 17, 18]. Therefore, the conditions of an analytical reaction should be carefully chosen to provide a compromise between the enzyme activity, the efficiency of the catalytic reaction, and the stability of the product formed.

Nowadays, the colloidal nanoparticles (NPs) composed of noble metals such as gold have gained great scientific attention due to their oxidoreductase-like activity (such NPs can mimic the horseradish peroxidase, glucose oxidase, or catalase) [19–21]. The superficial redox activity of gold NPs gives a number of possibilities of their use as enzyme mimics for the assay systems. The catalytic activities of NPS can be tailored according to the availability of substrates and the process conditions [2, 22, 23]. Increased attention has been paid to polymer-stabilized nanostructures characterized by the increased resistance to salt-induced aggregation or inactivation induced by extreme pH values. Improved stability of this type of NPs contributes to their catalytic activity, and it is crucial for further applications of nanozymes in complex media [24, 25]. In contrast to HRP, the catalytic activity of nanoparticles cannot be so easily inhibited by hydrogen peroxide, which allows the use of higher concentrations of this substrate in the catalysis medium. In addition, the low affinity of hydrogen peroxide to noble metal-based nanozymes reflects in the relatively high Michaelis constant values (*K*_M_), and this fact imposes the need of application of relatively highly concentrated H_2_O_2_ in solution for the efficient conversion of the substrates into colored products.

Despite mentioned differences in properties and the mechanism of activity, in a vast majority of the literature reports, the characterization methods of the catalytic properties of NPs have been adapted from the studies related to native enzymes [2, 5, 8, 9]. It should be pointed out that application of enzyme mimetics opens up new possibilities of the sample composition and the type and concentration of substrates. On the other hand, the significance of unusual composition of reaction media and the high concentration of H_2_O_2_ on the properties of the substrate–hydrogen peroxide redox couple remains in our opinion underestimated. Strongly oxidizing environment in combination with a low pH and the presence of some of components of samples (e.g., chloride anion) result in the formation of reactive species, which were described before the first nanomaterials were reported [26, 27]. Those compounds can interfere with the analytical reaction or interact with chromogenic substrates and applied catalysts [28]. To the best of our knowledge, no insight on such phenomena in case of nanozymes was performed. Therefore, careful studies concerning the influence of pH and composition of a sample on the generation of colored products from typical substrates (ABTS, TMB, OPD) by AuNPs were carried out in the framework of the presented work. Few improvements and restrictions in well-known analytical procedures regarding the sample composition and optima of pH for typical peroxidase substrates were proposed. A new insight on previously described procedures and their application in AuNP-mediated catalysis may be advantageous from the point of view of studies on oxidoreductase-like activities of nanozymes over a wide pH range.

## Materials and methods

### Reagents

Horseradish peroxidase type VI, from *Amoracia rusticana* (HRP, E.C.1.11.1.7, RZ > 2.5), gold(III) chloride trihydrate, sodium borohydride, 2,2′-azino-bis(3-ethylbenzothiazoline-6-sulfonic acid) diammonium salt (ABTS), 4-aminoantipyrine (4-AAP), phenol (PhOH), 3,3′,5,5′-tetramethylbenzidine (TMB), *o*-phenylenediamine dihydrochloride (OPD), bromopyrogallol red (BPR), and dimethyl sulfoxide were all purchased from Sigma Aldrich. Hydrogen peroxide (29.5 %, concentration determined spectrophotometrically at 240 nm) was purchased from POCh (Poland). Hyperbranched polyglycidol (HBPG, molecular weight ∼3.2 kDa) was prepared according to a previously presented procedure [29]. All reagents were used as received.

All glassware used for AuNP preparation were rinsed with *aqua regia* (concentrated hydrochloric acid/nitric acid 3:1 (*v*/*v*)) prior to use. Universal buffer (containing acetate, borate, and phosphate, each in concentration of 100 mM) was prepared by dissolution of appropriate amounts of acids in Milli-Q water and adjusted to desired pH using concentrated solution of sodium hydroxide. Other buffer solutions in concentrations of 200 mM (unless otherwise stated) were prepared in a similar way, and their pH was adjusted with the use of concentrated solution of sodium hydroxide (for phosphate, citrate, and MES buffers), hydrochloric acid (for glycine-HCl buffer), or phosphoric acid (for glycine-H_3_PO_4_ buffer), respectively. Hydrogen peroxide solutions were prepared daily by diluting the stock solution. Stock solution of TMB (20 mM) was prepared by dissolving the solid in dimethyl sulfoxide. Solutions of the remaining substrates in desired concentrations were prepared by dissolving the appropriate amounts of solids in Milli-Q water.

### Instrumentation

The UV-Vis spectra were recorded with the use of a Lambda 25 spectrophotometer (Perkin-Elmer) and quartz microcuvettes of 1 cm pathlength (Hellma Analytics). The average diameter of AuNPs was calculated based on TEM micrographs captured by a JEOL JEM-2100 transmission electron microscope. Comparative studies of catalytic activities were performed on a Sunrise Magellan microplate reader (TECAN Instruments) at room temperature using polystyrene 96-well plates.

### Preparation of HBPG-stabilized AuNPs

Five milliliters of HBPG aqueous solutions (0.5 mg mL^−1^) and a freshly prepared 10 mM aqueous solution of HAuCl_4_ (0.625 mL) were sealed and magnetically stirred for 10 min in a glass vial. Further, freshly prepared 50 mM aqueous solution of NaBH_4_ (0.625 mL) was injected to vigorously stirred solution of precursor and polymer. Then, AuNP solution was magnetically stirred in the absence of light for the next 24 h to finalize nanocrystal growth.

### Optical studies on formation and stability of OPD_ox_ and TMB_ox_ in buffered solutions

To 1.75 mL of buffered solution, 100 μL of stock H_2_O_2_ solution, and 50 μL of HBPG@AuNPs (C_Au_ = 1 mM) in quartz cuvette, 100 μL of TMB (20.0 mM) or OPD (20.0 mM) stock solution was added. For comparative purposes, the samples without the presence of hydrogen peroxide were examined. Immediately after mixing, a set of spectra in the range of 250–700 nm at 3-min intervals were registered.

### Determination of catalytic activity of AuNPs or HRP

The mixture of substrates (200 μL in each well) consisting of 500 mM H_2_O_2_ in case of nanozymes (except Michaelis-Menten kinetics studies) and 2.0 mM H_2_O_2_ in case of HRP and chromogenic substrates was as follows: (PhOH/4-AAP) 100.0 mM of phenol, 2.5 mM 4-AAP; (TMB) 1.0 mM of TMB; (ABTS) 1.0 mM of ABTS; (OPD) 2.0 mM of OPD; and (BPR) 0.1 mM BPR unless otherwise stated. Catalytic reactions were initialized by the addition of 50 μL of aqueous solutions of AuNPs (C_Au_ = 100 μM) or HRP (C = 0.05 μg mL^−1^). Optical pathlengths were normalized for total volume of 250 μL in case of each substrate.

Michaelis constants (*K*_M_) for hydrogen peroxide were calculated from apparent steady-state kinetic curves with the use of Lineweaver-Burk double reciprocal plots. For this purpose, a set of experiments in media differing in H_2_O_2_ concentration (from 25 to 1000 mM) were performed. Values of catalytic activity were based on initial, nearly linear slopes of kinetic curves recorded for the first 150 s of reaction.

## Results and discussion

The optical determination of analytes with the use of bioreceptors labeled with catalytic nanoparticles requires two steps of signal generation and its readout. The measured reaction rate is a result of several factors such as activity and concentration of catalyst, susceptibility of substrate to oxidation under applied conditions, and occurrence of side reactions, e.g., degradation of colored product or spontaneous, non-catalytic reactions. To guarantee the best analytical performance, it is desirable to maximize the efficiency of catalytic reaction while minimizing other non-catalytic processes, which may lead to false-positive results. This objective requires the careful examination of the impact of both reaction conditions and medium components on the formation of colored products. A number of studies on the development and characterization of nanoparticles (NPs) as horseradish peroxidase mimetics for bioanalytical systems have been published recently. However, in most cases, the activity measurements have been performed without paying excessive attention to fundamental differences in the structure, stability, and mechanism of action between nanozymes and HRP.

**Fig. 1:**
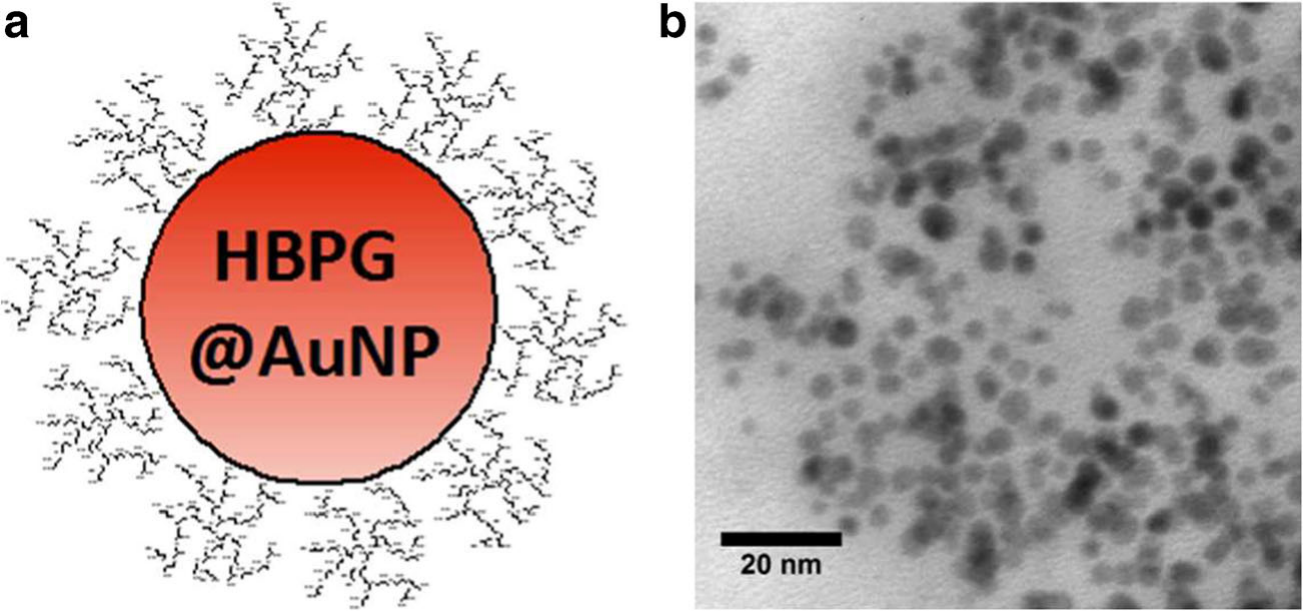
Scheme (**a**) and TEM image (**b**) of gold nanoparticles stabilized with hyperbranched polyglycidol (HBPG@AuNPs)

To investigate the properties of the most popular substrate systems, the highly active and stable gold nanoparticles coated with hyperbranched polyglycidol (HBPG@AuNPs) were chosen and applied as a model nanozyme. The illustrative scheme and TEM micrograph of the examined nanoparticle are depicted in Fig. 1. The average diameter of nanoparticles used amounted to 5.0 ± 0.8 nm (based on TEM measurement).

### Influence of a buffer and its pH for oxidation of ABTS

**Fig. 2:**
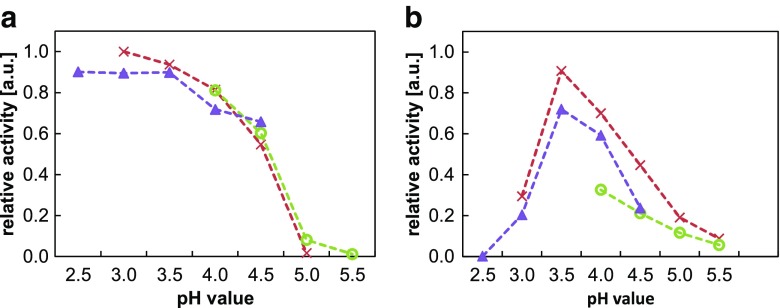
Effect of a buffer and its pH on the formation of ABTS anion radical in the reaction catalyzed by HBPG@AuNPs (**a**) and HRP (**b**). Relative activities were normalized to the maximum of initial absorbance slope for each catalyst. All final buffer concentrations amounted to 136 mM. () phosphate, () citrate, () MES

The characterization of catalytic properties for peroxidase mimetics requires the use of stable chromogen, which is able to generate a colored product over a wide range of pH. ABTS as a popular and sensitive peroxidase substrate of anionic character, widely used for the studies of substrate-NPs surface interactions and applied in bioanalysis, was one of our choices. The activity of HBPG@AuNPs and native HRP against ABTS in a series of buffers of different pH was examined and compared. As noticed, ABTS showed a significant dependence of efficiency in generating a chromogenic product on a pH of applied medium, exhibiting the highest reaction rate in the acidic range. As shown in Fig. 2, the relation of the catalytic reaction rates and pH was characterized by a similar course, regardless of the type of catalyst applied (except very acidic media, where protein inactivation occurs). The optimum performance for AuNPs against ABTS was observed for solutions of pH no higher than 3.0. In contrast to the enzyme, the use of nanozymes enables extending the applicability of ABTS for media of low pH. This indicates that efficient transformation of ABTS in acidic medium is the individual feature of this substrate rather than changes in activity of the catalyst. It should be underlined that a substantial decrease in the efficiency of colored product formation for samples of pH higher than 4.5 arises from relatively high redox potential of the substrate system resulting in a relatively low susceptibility to oxidation [2], and this does not point out the changes in the activity of the catalyst applied.

In addition to the apparent inactivation, HRP also showed a significant dependence on the type of buffer applied. Its highest activity was noticed in buffers containing organic anions such as citrate or acetate, while a solution containing phosphate decreased the enzyme activity. Low sensitivity of ABTS assay in Good’s buffers (such as MES) could be assigned to the degradation of colored radical (product of oxidation) in a contact with buffering species in samples of low H_2_O_2_ concentration (in comparison to the samples applied for nanozyme). The mentioned phenomenon results in the widespread use of citrate and acetate buffers in protocols regarding studies on activity of nanozymes, while the other tested buffers can be successfully used for this aim because their influence on the relative activity of AuNPs is generally negligible (see Fig. 2b).

### Interferences of chloride in ABTS assay

On the basis of the studies described above, the applicability of ABTS as a hydrogen donor for activity studies of NPs in acidic media was confirmed. However, further studies demonstrated a significant interference arising from the presence of chloride, which is very common in real samples and can be used as a component of buffers (Gly-HCl buffer tested).

**Fig. 3:**
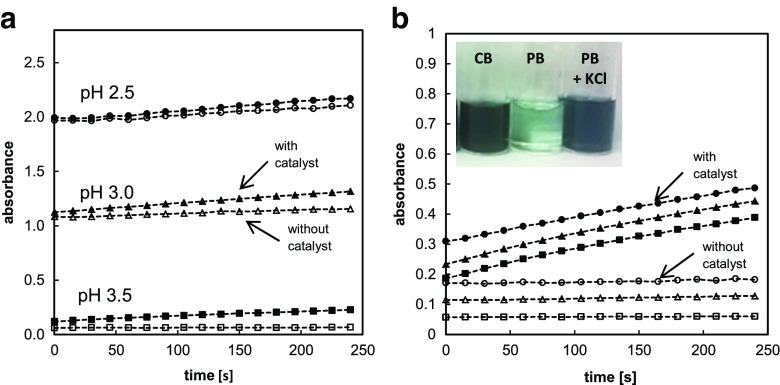
Effect of non-catalytic oxidation of ABTS on absorbance slopes at selected pHs in a glycine-HCl buffer (**a**) and in a glycine-H_3_PO_4_ buffer (**b**). pH 2.5 (*filled circles*), pH 3.0 (*filled triangles*), pH 3.5 (*filled squares*). *Empty bars* represent corresponding samples without HBPG@AuNPs. *Inset*: effect of addition of ABTS (2 mM) to solutions of selected buffers containing 500 mM of H_2_O_2_. Last sample (*right*) additionally contains 50 mM of KCl. *PB* phosphate buffer, *CB* citrate buffer, both of pH 2.0. Picture was taken after 1 h

As observed, the high concentration of H_2_O_2_ in a solution of glycine-HCl buffer resulted in spontaneous oxidation of ABTS without the presence of a catalyst. Non-catalytic side reaction intensified with the increasing acidification of the sample and resulted in a significant and undesirable effect (Fig. 3). At pH 3.0, the slope of the background absorbance (sample without a catalyst) amounted to more than 47 % of the total slope of the reaction catalyzed by AuNPs, which makes the study of catalytic reaction practically impossible to carry out under such conditions. The explanation of observed phenomenon covers the oxidation of chloride by means of hydrogen peroxide, which results in a generation of reactive chlorine species such as hypochlorous acid or chlorine oxides acting as aggressive oxidants [26, 27]. These species promote non-catalytic oxidation of ABTS to colored cationic radical (ABTS^●+^) according to reactions presented below [30].

1 $$ {\mathrm{H}}^{+} + {\mathrm{Cl}}^{-} + {\mathrm{H}}_2{\mathrm{O}}_2\to\ \mathrm{HClO} + {\mathrm{H}}_2\mathrm{O} $$

2 $$ \mathrm{HClO} + \mathrm{ABTS} + {\mathrm{H}}^{+}\to\ {\mathrm{Cl}}^{-} + 2\ {\mathrm{ABTS}}^{\bullet +} + {\mathrm{H}}_2\mathrm{O} $$

To confirm the mentioned mechanism, comparative studies in buffer solutions of corresponding pH values, but devoid of chloride (sodium phosphate and glycine-phosphate buffer), were performed, and no adverse effect was noticed.

A second notable observation was the positive effect of phosphate-based buffers on the suppression of ABTS autoxidation. The experiment conducted in various buffers of pH 2.0 (0.2 M phosphate, mixed 0.1 M phosphate + 0.1 M citrate and 0.2 M citrate) revealed that the substrate dissolved in phosphate-containing solutions exhibits a significantly lower rate of spontaneous oxidation in comparison to citrate buffer (see inset of Fig. 3b). Therefore, for the evaluation of catalytic activity of AuNPs, the application of phosphate buffer or phosphate-containing buffers (phosphate-citrate or phosphate-acetate) is strongly recommended.

### Oxidation of TMB and OPD in the acidic media and the influence of chloride

**Fig. 4:**
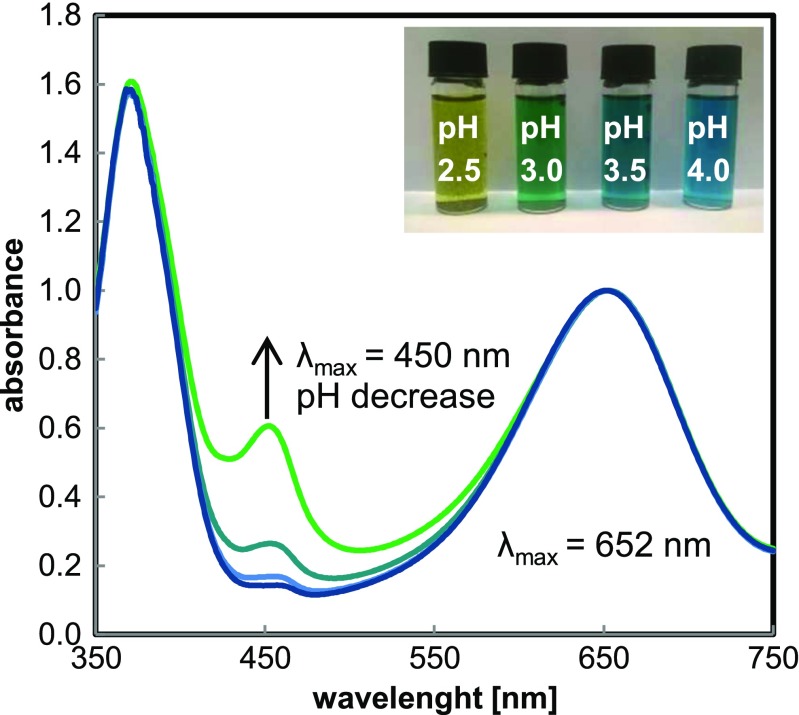
UV-Vis absorption spectra of TMB oxidation products in phosphate buffer of various pH, () 4.0, () 3.5, () 3.0, () 2.5. Spectra were normalized at 652 nm. *Insert* shows image of examined samples after 15 min of reaction

The most widely used substrate for the preliminary characterization of peroxidase mimetics is 3,3′,5,5′-tetramethylbenzidine. However, the blue charge transfer complex, which is a product of single-electron oxidation of TMB, is quite unstable in acidic media and susceptible to further oxidation to diimine (yellow product). This fact can strongly affect the obtained optical signal [31]. Moreover, mechanism of charge transfer complex generation involves the presence of unoxidized substrate (in equilibrium with cationic radical); therefore, the high conversion of TMB during catalytic reaction disturbs the analytical signal, which is often overlooked in TMB-based assays. To minutely examine the mentioned phenomenon, the effect of catalytic reaction on the equilibrium of diimine–charge transfer complex for high molar excess of H_2_O_2_ (500× compared to TMB) was examined. It was noticed that for both examined buffers (phosphate, Gly-H_3_PO_4_), the ratio of diimine (yellow, *λ*_max_ = 450 nm) to charge transfer complex form (blue, *λ*_max_ = 652 nm) is pH-dependent and it increases while decreasing the pH of a sample, which was manifested as a gradual change of reaction mixture into yellow through green color (see Fig. 4).

A key issue in ensuring the stability of the blue complex was to maintain a sufficiently high concentration of unoxidized TMB. Based on conducted experiments, it was found that the concentration of TMB, which assures stable *A*_652_/*A*_450_, is 1.0 mM (it was confirmed by a set of spectra registered every 3 min during the first 24 min). In such case, a small degree of chromogen conversion provides apparent steady-state conditions, which assure equilibrium between cationic radical and charge transfer complex.

Test conducted for solutions of pH 2.5 containing different concentrations of TMB (0.5 and 1.25 mM) confirmed that for lower concentration of TMB, the equilibrium was shifted towards the formation of diamine, which was generated at the expense of the blue product. It reflected as the ratio of *A*_652_/*A*_450_, which amounted to 1.64. In turn, for higher concentration of TMB, *A*_652_/*A*_450_ ratio amounted to 2.66. When increasing the TMB concentration (from 0.5 to 1.25 mM) in a solution of ongoing catalytic reaction by addition of the substrate, the immediate increase of absorbance at 652 nm was observed, which confirmed the shift in the equilibrium towards charge transfer complex. Similar conclusions can be drawn for OPD, for which the generation of product 2,3-diaminophenazine derivatives also involves the presence of unoxidized amine [32].

**Fig. 5:**
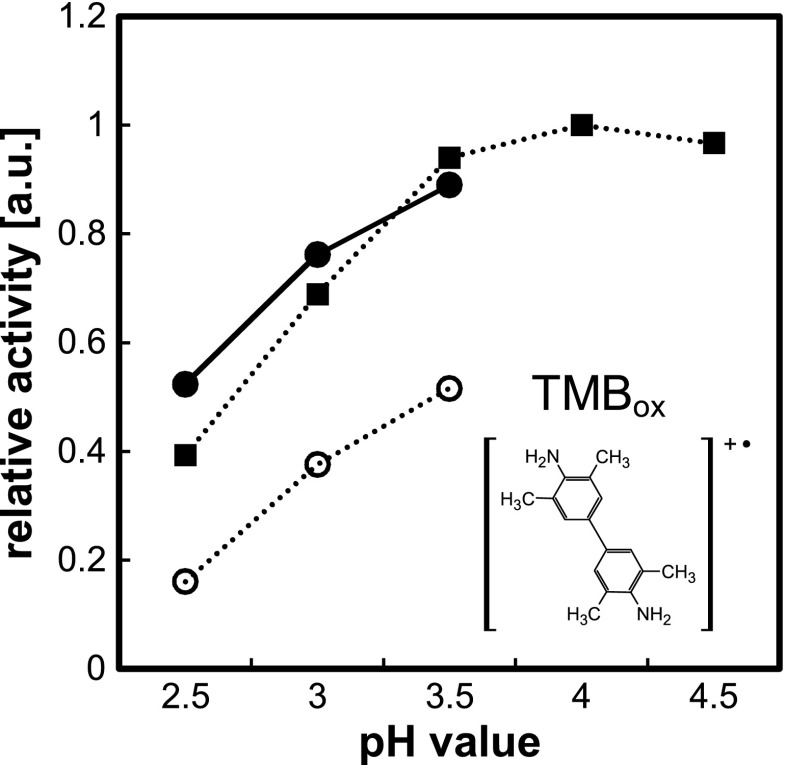
Effect of a buffer and its pH on the TMB oxidation to blue charge transfer complex (measured at 652 nm) in the reaction catalyzed by HBPG@AuNPs. Relative activities were normalized to the maximum of initial absorbance slope. All final buffer concentration amounted to 136 mM. Sodium phosphate (*filled squares*), glycine-H_3_PO_4_ (*filled circles*), glycine-HCl (*open circles*). *Inset*: structure of 3,3′,5,5′-tetramethylbenzidine cationic radical

The acid-dependent reactivity of TMB and OPD results from their limited solubility in aqueous solutions, which is improved due to the protonation of one of amino group of these substrates [33]. However, the excessive lowering of pH results in protonation of both amino groups, and it makes the substrate insusceptible to catalytic oxidation, which explains the apparent decrease in the reaction rate. Therefore, the mentioned substrates exhibit maxima of reaction efficiency over the pH range of 4.0–5.0, although some studies of nanoparticle activity under more acidic pH were also reported [34]. As depicted in Fig. 5, aromatic amines (the same trends were observed for TMB and OPD) may be utilized as substrates in very acidic media (pH 2.5–3.5), but such systems are characterized by a lower sensitivity and lower product stability than in the case of pH ranging from 4.0 to 5.0.

As indicated in the paragraph above, the presence of chloride may substantially interfere with catalytically induced redox reactions under acidic conditions. Hence, the influence of selected buffers on the oxidation of TMB and OPD was examined, and the results for TMB are shown in Fig. 5. In the case of TMB and OPD, utilization of Gly-HCl buffer leads to a substantial decrease of colored product formation rate in highly acidic media (pH 2.5–3.5) compared to other applied buffers. In contrast to ABTS-based assay, non-catalytic oxidation of chromogenic compounds was not observed; only the formation of colored products was significantly suppressed. The low rate of blue complex formation in buffers containing chloride, however, was not the result of its direct oxidation to diimine described above.

### Effect of hydrogen peroxide concentration

As reported in previous chapters, the strongly oxidizing environment (concentration of H_2_O_2_ about two orders of magnitude higher than in the case of peroxidase), which is applied for activity studies of nanozymes, may entail new, previously unknown complications. The decrease of hydrogen peroxide concentration without a significant loss of catalytic activity could minimize the risk of side effects described above. For this purpose, the effect of pH and various substrates on a thermodynamic affinity of hydrogen peroxide to gold nanoparticles, expressed as value of Michaelis constant (*K*_M_), was examined. For the determination of *K*_M_, except the discussed OPD, TMB, and ABTS, also substrates capable of operation at neutral or slightly basic pH such as phenol coupled with 4-aminoantypirine and bromopyrogallol red were used.

Based on the relation of chromogen oxidation rates and concentration of H_2_O_2_, Michaelis constants for each substrate at optimum pH were calculated with the use of double-reciprocal plots. The high *K*_M_ values for HBPG@AuNPs (representing low affinity of H_2_O_2_ to NPs) characterized substrates working in acidic media—TMB and ABTS (see Table 1).

**Table 1:** Values of Michaelis constants representing affinity of H_2_O_2_ to HBPG@AuNPs determined with the use of various hydrogen donors at optimal pH values

Hydrogen donor	*K*_M_ (mM)	Medium pH
ABTS	777.8 ± 9.3	3.0
TMB	682.8 ± 14.3	4.5
OPD	205.8 ± 17.9	5.0
BPR	62.80 ± 6.34	6.5
PhOH	109.0 ± 4.7	8.5

As it was generally observed, the application of the most common chromogens (ABTS, TMB, OPD) in combination with Au-based nanozymes require, for their optimum performance, about 2- to 4-fold higher levels of H_2_O_2_ than for the phenolic derivatives. The observed phenomenon may be attributed to both different stabilities of obtained HO^●^ as well as different oxidation potentials of chromogens.

**Fig. 6:**
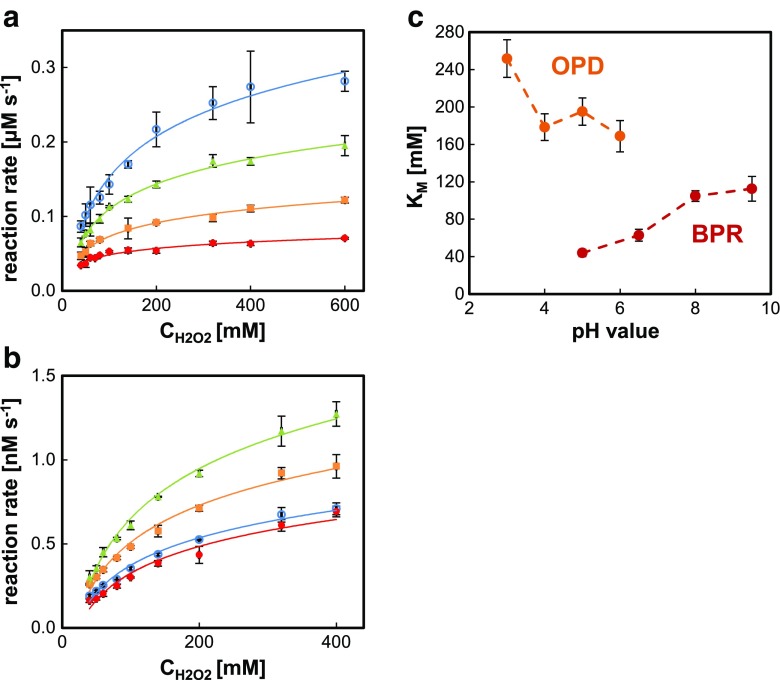
Dose-response curves for oxidation of BPR (**a**) and OPD (**b**) at various pH values of universal buffer, BPR: () 5.0, () 6.5, () 8.0, () 9.5; OPD: () 3.0, () 4.0, () 5.0, () 6.0. Concentration of BPR, OPD, and HBPG@AuNPs were fixed at 80 μM, 1 mM, and 20 μM (expressed as gold precursor), respectively. Influence of medium pH on *K*_M_ values against hydrogen peroxide for oxidation of BPR and OPD catalyzed by HBPG@AuNPs (**c**). The *error bars* represent the standard deviation of three measurements

According to the mechanism of heterogeneous catalysis at AuNP active sites, differences in *K*_M_ towards H_2_O_2_ should be substantially dependent on pH. The surface of nanoparticles is prone to adsorption of protons and hydroxyl anion, which can significantly change the character of the active sites and affect the performance and even the nature of the conducted catalysis [23]. In order to evaluate the influence of pH on hydrogen peroxide affinity to the applied catalyst, a series of *K*_M_ values for the same hydrogen donor at different pH were calculated. Two types of substrates—aromatic amine (OPD) and phenol derivative (BPR)—working in slightly different pH ranges were chosen (see Fig. 6).

The results obtained for BPR indicated that the pH of the catalysis medium only slightly affects the affinity of H_2_O_2_ to gold nanoparticles. HBPG@AuNPs exhibit monotonic increase of *K*_M_ when increasing pH for BPR oxidation, which represents the decrease of H_2_O_2_ affinity as depicted in Fig. 6c. Surprisingly, the opposite trend was observed for OPD, which indicates that the relation between *K*_M_ and pH towards hydrogen peroxide is an intrinsic feature of applied substrate system. After a change of co-substrate to phenol, relations of Michaelis constants for H_2_O_2_ at different pHs remain similar to BPR. The results show that *K*_M_ of the peroxidase mimics are determined by the substrate applied and to a lesser extent by the pH of the reaction medium.

As shown, change of the pH of the reaction medium has a relatively small effect on the affinity of hydrogen peroxide to active sites of the catalyst. Therefore, in order to ensure the efficient NP-mediated catalysis at relatively low concentrations of H_2_O_2_, careful selection of hydrogen donor seems to be the most effective strategy.

## Conclusions

This work underlines the potential problems that can be encountered when applying nanozymes of HRP-like activity for oxidation of well-known peroxidase substrates such as ABTS, OPD, or TMB. It presents a set of useful strategies for reliable study of catalytic activity of peroxidase mimetics in media of high hydrogen peroxide concentration and over a wide pH range. The presented results should be of special importance for those who characterize a new generation of robust catalytic nanoparticles and apply them in bioanalysis.

In the reference to ABTS-based assays, the following conclusions and guidelines can be presented:

- AuNPs exhibit HRP-like activity in ABTS assay in samples of low pH, which is unattainable for the native enzyme.
- To maximize the formation of ABTS oxidation product with the use of AuNPs, a medium of pH lower than 3.0 should be applied and the presence of chloride in sample should be avoided then.
- Among tested buffer solutions, phosphate buffer provides the highest stability of ABTS, and therefore it is recommended for AuNP-based assays.

In the case of TMB- and OPD-based assays, the following conclusions and guidelines can be presented:

- Application of TMB- and OPD-based assays in media of pH below 3.0 is not recommended due to relatively low sensitivity. If such pH is to be applied, ABTS should be chosen as a substrate.
- If application of TMB or OPD in media of pH below 4.0 is necessary, the presence of chloride should be avoided due to interferences, which cause a deterioration of the assay’s sensitivity.
- To enhance the stability of charge transfer complex at pH below 4.0, the relatively high concentration of substrate should be introduced (higher than 1 mM). The lower the pH of the medium, the higher concentration of free substrate is necessary to suppress further oxidation to diamine.
- Stopping the oxidation of TMB or OPD through the acidification of reaction medium (to pH below 1, which is often used in routine analyses with HRP-labeled receptors) is ineffective in case of AuNPs due to the incomplete inactivation of the catalyst.

In reference to H_2_O_2_, the following conclusions can be drawn:

- A careful selection of hydrogen donor used in HRP-like activity assay allows for the reduction of the hydrogen peroxide concentration, which is necessary for efficient catalysis (due to differences in *K*_M_).
- A change of medium pH (using the same hydrogen donor) influences the affinity of hydrogen peroxide to AuNPs to a lower extent than a change of co-substrate.
- Less common substrates, capable of working at near-neutral pH, such as phenolic compounds (PhOH, BPR) can become attractive agents for the examination of nanozyme activity due to lower concentrations of H_2_O_2_ required for the efficient catalysis in comparison to popular, acid-dependent substrates (like OPD, TMB, or ABTS).
